# Over the River

**Published:** 1864-01

**Authors:** N. A. W. Priest


### OVER THE RIVER.

BY MISS N. A. W. PRIEST.

OVER the river they beckon to me,
Loved ones who've crossed to the further side,
The gleam of their snowy robes I see,
But their voices are lost in the dashing tide.
There's one with ringlets of sunny gold,
And eyes the reflection of heaven's own blue,
He crossed in the twilight gray and cold,
And the pale mist hid him from mortal view;
We saw not the angels who met him there,
The gates of the city we could not see.
Over the river, over the river,
My brother stands waiting to welcome me.

Over the river the boatman pale
Carried another, the household pet;
Her brown curls waved in the gentle gale,
Darling Minnie! I see her yet.
She crossed on her bosom her dimpled hands,
And fearlessly entered the phantom bark,
We felt it glide from the silver sands,
And all our sunshine grew strangely dark;
We know she is safe on the further side,
Where all the ransomed and angels be;
Over the river, the mystic river,
My childhood's idol is waiting for me.

For none return from those quiet shores,
Who cross with the boatman cold and pale;
We hear the dip of the golden oars,
And catch a gleam of the snowy sail;
And lo! they have passed from our yearning hearts,
They cross the stream and are gone for aye.
We may not sunder the vail apart
That hides from our vision the gates of day.
We only know that their barks no more
May sail with us o'er life's stormy sea;
Yet somewhere I know on the unseen shore,
They watch, and beckon, and wait for me.

And I sit and think, when the sunset's gold
Is flushing river and hill and shore,
I shall one day stand by the water cold
And list for the sound of the boatman's oar;

I shall watch for a gleam of the flapping sail,
  I shall hear the boat as it gains the strand;
I shall pass from sight with the boatman pale,
  To the better shore of the spirit-land.
I shall know the loved who have gone before,
  And joyfully sweet will the meeting be,
When over the river, the peaceful river,
  The Angel of Death shall carry me.

### HEAVEN.

BY MISS N. A. W. PRIEST.

